# Expression of *Ki-67* and *Βeta-Catenin* in Pseudoepitheliomatous Hyperplasia and Squamous Cell Carcinoma in Oral Mucosal Biopsies : An Immunohistochemical Study

**DOI:** 10.31557/APJCP.2020.21.1.157

**Published:** 2020

**Authors:** Bismah Ahmad, Mohammad Asif, Shahid Jamal, Muhammad Zaib Khan, Mohammad Tahir Khadim

**Affiliations:** 1 *Department of Histopathology, Armed forces Institute of Pathology (AFIP), *; 2 *Department of Histopathology, Watim Medical and Dental College,*; 3 *Department of Endodontics , Margalla Institute of Health Sciences, Rawalpindi, Pakistan. *

**Keywords:** Squamous cell carcinoma, Pseudoepitheliomatous hyperplasia, Beta-catenin, Ki-67

## Abstract

**Objective:**

To examine the immunohistochemical expression of *Ki-67* and *beta-catenin* in pseudoepitheliomatous hyperplasia and squamous cell carcinoma (SCC) in oral mucosal biopsies.

**Methods:**

In this comparative cross sectional study, 70 cases of each PEH and OSCC were taken from the patients of both genders and in all age groups. Study was conducted at Armed Forces Institute of Pathology (AFIP), Rawalpindi from Dec 2017 to March 2019. Statistical analysis was done with the help of SPSS Version 24.0. We used Chi-Squared test with p value of < 0.05 which was considered as statistically significant.

**Results:**

In the current study, 80 (57.1%) male and 60 (42.8%) female patients with the mean age of 51.69 ± 16.121 (mean ± SD) years were included. It was found that 6-25% *Ki-67* labeling index was observed in all (70) PEH cases, which involved only basal layer of the epithelium. Whereas, *Ki-67* labeling index was highly expressed in tumor of high grade malignancy than tumor of low grade malignancy. On the other hand, expression of membranous *beta-catenin* was higher in PEH and cytoplasmic *beta-catenin* expression was higher in OSCC.

**Conclusion:**

It is concluded that *Ki-67* and *beta-catenin* showed significant expression in PEH and OSCC in oral mucosal biopsies especially those with intense inflammation or unoriented tissue, helping the clinicians to arrive at a final diagnosis before planning any surgical intervention.

## Introduction

Oral cancer is an important problem of global public health, constituting approximately 5% of all cancers (Vig et al., 2015). Out of this 5%, almost 90% constitutes oral squamous cell carcinoma. In Pakistan, it accounts for 10% of all malignancies (Wahid et al., 2005). According to Pakistan Medical Research Council (PMRC), OSCC accounts for the majority of the cancers among males than in females because of heavier consumption of smoked tobacco and alcohol in males with tongue and lips are the most frequent occupied sites (Jamal et al., 2006). The major risk factor for OSCC is use of tobacco which may be in the form of cigarettes, cigars, pipes and chewing betel nuts. Other risk factors include heavy consumption of alcohol, nutritional deficiencies, sepsis, genetic mutation involving p53 gene, human papillomavirus (HPV) infection, Epstein-Barr virus (EBV) infection and poor dental hygiene (Markopolous, 2012). These risk factors leads to changes in epithelium of oral mucosa causing transformation of normal keratinocytes into abnormal keratinocytes, squamous cell atypical and invasive features (Feller et al., 2013).

One of the benign mimics of OSCC is pseudoepitheliomatous hyperplasia (PEH) which is defined as a histopathological response to numerous stimuli, such as shock, disease, irritation and neoplasia (Zayour and Lazova, 2011). Histologically, it is seen as tongue like epithelial propagations penetrating into connective tissue (Zarovnaya and Black, 2005). When the mucosal biopsies are of small size, ulcerated, unoriented and inflamed or when the profound rete pegs are cut obliquely, it become extremely difficult to differentiate between PEH and OSCC. Hence, it is important to differentiate between PEH and OSCC as the whole treatment plan of the surgeons and oncologists relies on correct diagnosis (Nayak et al., 2015).

*Ki-67* is a monoclonal antibody for proliferation. It is strictly associated with cell proliferation and aggressiveness of malignant tumours (Dadfarnia et al., 2012). During cell cycle, the presence of *Ki-67* protein is seen during prophase and metaphase (G1, S, G2 and M phase) but is absent during resting cells (G0) (Scholzen et al., 2000). Whereas; *beta-catenin* is a subunit of cadherin protein complex. *beta-catenin* is a 92 kDa protein normally found in cytoplasm of the cell in sub-membranous location which participate in regulation and organization of cell–cell adhesion and transcriptional regulator (Liu at al., 2010). Mutation or overexpression in the *beta-catenin* gene results in nuclear accumulation of the protein. Reduced expression of membranous *beta-catenin* and amplified expression of cytoplasmic / nuclear *beta- catenin* has been associated with increase grading and invasiveness of tumor (Rosado et al., 2013).

So far, the expression of *Ki-67* and *beta-catenin* protein has not been completely explained in human PEH and OSCC samples by immunohistochemistry (IHC). The aim of this study was to examine the expression of *Ki-67* and *beta-catenin* by IHC in paraffin-embedded oral mucosal tissues exhibiting PEH and OSCC.

## Marterials and Methods

An institutional ethical committee approval (IRB, AFIP) (letter no. MP-ORP16-10/READ-IRB/17/394) was taken before the start of the study. A total of 140 oral mucosal biopsy samples, 70 cases each of PEH and OSCC, along with normal mucosal biopsy as control group were collected from Armed Force Institute of Pathology (AFIP), Rawalpindi, Pakistan from December 2017 to March 2019. 

Along with clinical histories of each case, the data of age, gender and site were noted. Immunohistochemistry was performed on 140 mucosal biopsies along with adjacent controls. Scanty and poorly fixed specimens were excluded from the study. Antibodies used for IHC were: monoclonal rabbit *Ki-67* (clone no. EP5, catalogue no. BSB 5709, ready to use) from Bio SB (Santa Barbara, CA, USA); and monoclonal mouse *beta-catenin* (clone no. 17C2, catalogue no. PA0083, ready to use) from Leica Bio system (Newcastle, UK). Results were interpreted on light microscope (Binocular Olympus (Tokyo, Japan) Model CX-21) using high power field objective (10x, 40x) and further counter checked by consultant pathologist. Evaluation of *Ki-67* and *beta-catenin* was performed as follow:

The intensity of brown colored nuclear *Ki-67* staining which is confined to spinous layer or both basal and parabasal layer or only basal layer of epithelium is graded as (Humayun et al., 2011) : 

Mild……. +light brown color

Moderate……. ++ dark brown color

Severe……… +++ very dark brown color 

The pattern of staining was assessed in percentage by calculating the positive cells per 100 basal cells, parabasal or spinous cell layer of epithelium. The percentage of positive cells or labeling index (LI) was as follows (Humayun et al., 2011): 

Negative = 0-5%

basal layer staining = 6-25%

basal and parabasal layer staining = 26-60%

basal, parabasal and spinous layer staining = 61-99%.

On the other hand, *beta-catenin* positive cell is defined as brown staining of nucleus, cytoplasm or cell membrane of epithelium and is expressed in the form of immunoreactivity as follows (Zaid et al., 2015):

The proportion score (P) was interpreted as:

0……………. Negative

1……………. <10% positive cells

2……………. 10-50% positive cells

3……………. 50-80% positive cells

4……………. >80% positive cells

The intensity score (I) was defined as: 

0……………. Negative / No staining 

1……………. Weak staining

2……………. Moderate staining

3……………. Strong staining

Immunoreactive combined score (T) = proportion score (P) x intensity score (I).

Immunoreactivity scores (T) of *beta-catenin* were categorized into three groups based on the final score:

Score 0……………. Negative

Score 1-4……………. Weak Staining

Score > 4……………. Strong Staining

## Results

In a total of 140 cases, 70 (50%) cases showed positive stained cells ranging from 6-25% Ki -67 labeling index. Whereas, 24 (17.1%) and 46 (32.9%) cases showed positive stained cells ranging from 26-50% and 51-99% Ki -67 labeling index respectively (Table 1).

Among 70 cases of PEH, all 70 (100%) cases showed positive staining with *Ki-67* antibody in the basal layer of the epithelium. The labelling index of *Ki-67* for PEH is 6-25%. 

**Figure 1:**
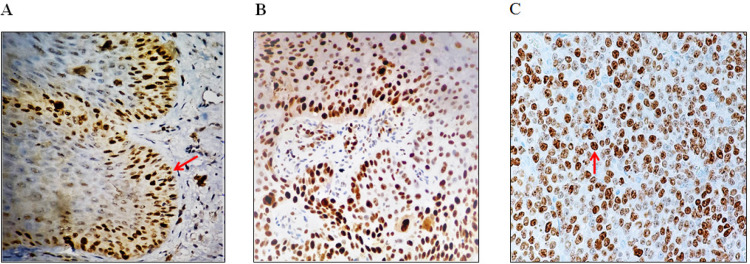
Expression of *Ki-67* in Different Oral Mucosal Regions; (A) *Ki -67* in PEH showing nuclear staining limited to basal layer (red arrow); (B) Ki-67 in WDOSCC showing 26-50% of dispersed positive nuclei; (C) *Ki-67 *in PDOSCC showing numerous intense (51-99%) scattered positive nuclei (immunohistochemistry staining, original magnification 400x).

Out of 70 cases of OSCC, 18 (45%) and 22 (55%) cases of well-differentiated OSCC (WDOSSC) showed *Ki-67* labelling index of 26-50% and 51-99 % respectively. No staining pattern of WDOSCC was observed between *Ki-67* labelling index of 6-25%. Similarly, 6 (22%) and 21 (77.8%) cases of moderately differentiated OSCC (MDOSCC) showed *Ki-67* labelling index of 26-50% in cases and 51-99 % respectively. While, no staining pattern of MDOSCC was observed between *Ki-67* labelling indexes of 6-25%. Interestingly, poorly differentiated OSCC (PDOSCC) showed only 51- 99 % *Ki-67* labelling index in 3(100%) cases. Whereas, no staining pattern was observed between index of 6-25% and 26-50% in PDOSCC (Figure 1).

Among 140 cases, score 0 was not found in any single case. Whereas, 52(37.1%) cases showed weak staining and 88(62.9%) cases showed strong staining (table 2).

Out of 140 cases (Table 3), 78 cases of the oral mucosal biopsies exhibited membranous expression of β- catenin in which 7.7% of membranous β- catenin was expressed in WDOSCC, 2.6% in MDOSCC, 0% in PDOSCC. In PEH, 87.7% of membranous β- catenin was expressed.

**Figure 2:**
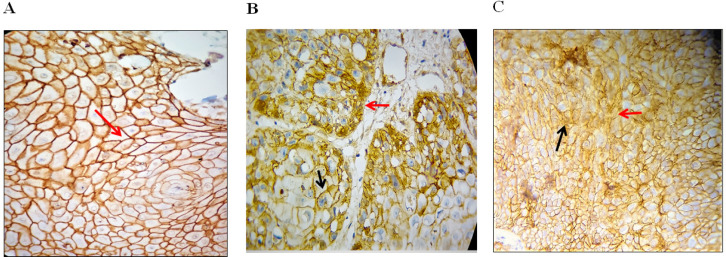
Expression of Beta-Catenin in Different Oral Mucosal regions; (A), Beta - catenin in PEH showing intense membranous staining (red arrow) throughout the epithelium; (B), Beta - catenin in WDOSSC showing membranous (black arrow) and cytoplasmic (red arrow) staining; (C)Beta-catenin in PDOSCC showing dispersed altered cytoplasmic (black arrow) and nuclear staining (red arrow) throughout the cancerous islands (immunohistochemistry staining, original magnification 400x).

Twenty-five (17.9%) cases of the epithelial cells exhibited cytoplasmic expression of β- catenin in which 56% of cytoplasmic β- catenin expression was seen in WDOSCC, 44 % in MDOSCC, 0% in PDOSCC and PEH. Only 1 case of the PDOSCC exhibited nuclear staining of *β- catenin*. But since, in this case, a little bit of cytoplasmic staining was also found, so, this case was included in cytoplasmic/nuclear expression. Eighteen cases of the oral mucosal biopsies exhibited membranous / cytoplasmic expression of *β- catenin* in which 64.3% of membranous / cytoplasmic *β- catenin* was expressed in WDOSCC, and 25.7% in MDOSCC. No membranous *β- catenin* expression found in PDOSCC and PEH. Nine cases of the oral mucosal biopsies exhibited cytoplasmic / nuclear expression of *β- catenin* in which 12.5% of cytoplasmic / nuclear *β- catenin *was expressed in WDOSCC, and 50% in MDOSCC. 37.5% in PDOSCC. In PEH, no cytoplasmic / nuclear* β- catenin* was expressed (Fig. 2). So, the statistically significant relation was observed between pattern of β-catenin expression and the histological diagnosis i.e. OSCC and PEH of p = .000 (p<0.05) .

**Table 1:** Expression of *Ki-67* in OSCC and PEH (n = 140).

*Ki-67* expression	Histological Diagnosis			
	PEH n (%)	WDOSCC n (%)	MDOSCC n (%)	PDOSCC n (%)
( 6 - 25%) n =70	70 (100%)	0 (0%)	0 (0%)	0 (0%)
( 26 - 50%) n =24	0 (0%)	18 (45%)	6 (22.2%)	0 (0%)
( 51 - 99%) n =46	0 (0%)	22 (55%)	21 (77.8%)	3 (100%)
Total n = 140(%)	70 (50%)	40 (28.6%)	27 (19.3%)	3 (2.1%)

**Table 2:** Patterns of Beta-Catenin Expression in PEH and OSCC (n = 140).

Pattern of Beta-catenin	Histological Diagnosis				
	PEH n (%)	WDOSCC n (%)	MDOSCC n (%)	PDOSCC n (%)	Total (140)
Membranous	70 (89.7%)	6 (7.7%)	2 (2.6%)	0 (0%)	78
Cytoplasmic	0 (0%)	14 (56%)	11 (44%)	0 (0%)	25
Membranous / Cytoplasmic	0 (0%)	18 (64.3%)	10 (35.7%)	0 (0%)	28
Cytoplasmic / Nuclear	0 (0%)	1 (12.5%)	5 (50%)	3 (37.5%)	9

## Discussion

Oral squamous cell carcinoma is the most frequent malignant neoplasm of oral region (Vig, 2015). There are a lot of structural features which help in differentiating oral squamous cell carcinoma from pseudoepitheliomatous hyperplasia on H / E sections. But, there has always been argument whether the mucosal biopsies are diagnosed as OSCC or PEH, when they are of small size, ulcerated, unoriented and inflamed or when the profound rete pegs are cut obliquely, as this will lead to unnecessary removal of tissue or additional therapy such as chemo radiotherapy (Zarovnaya and Black, 2005). 

Current study investigated the immunohistochemical expression of *Ki-67* proliferative marker and *beta-catenin* on pseudoepitheliomatous hyperplasia and PEH. 

In our study, PEH showed positive staining with *Ki-67* antibody in the basal layer of the epithelium with a labelling index of 6-25% while in most of the cases of OSCC; the entire epithelium showed continuous and prominent *Ki-67* labelling. The results are similar to the study made by Humayun and Prasad, (2011). which showed that *Ki-67* labelling index in WDOSCC is low (28 ± 20.82) while in MDOSCC and PDOSCC a significantly higher number of *Ki-67* positive cases with a mean of 47 ± 6.3 ( mean ± SD ) and 60.3 ± 2.8 ( mean ± SD ) respectively were seen. Similarly, in another study by Ashraf and his co-workers (Ashraf et al., 2010) studied 54 patients showing Ki- 67 staining increases as the tumor leads towards its severe stage.

Moreover, in a study of Finland by Luukkaa showed that the *Ki-67* and p53 as tumor markers and revealed that in PDOSCC, *Ki-67* expression was more diffuse and intense as the cells were less differentiated than WDOSCC and MDOSCC (Luukkaa et al., 2006). Similar finding have been seen in our study in which all PDOSCC cases showed 51-99% *Ki-67* expression range implying that high grade tumor showed great proliferative activity which has a poor prognosis.

On the other hand, beta- catenin is a protein normally found in cytoplasm of the cell in sub-membranous location which participate in regulation and organization of cell–cell adhesion and transcriptional regulator (Purcell et al., 2011). In a research carried out in China, Jiang and his fellows (Jiang et al., 2004) showed that increased membranous appearance of *beta-catenin* antibody was seen in pseudoepitheliomatous hyperplasia exhibiting good prediction of recovery- of the patient. Likewise, in the study by purcell (Purcell et al., 2011) illustrated that when there was increased expression of cytoplasmic/nuclear *beta-catenin* in squamous cell carcinoma of pharynx, the prognosis is poor. This was parallel to the present study in which all PDOSCC cases showed cytoplasmic/nuclear staining. 

In the present study, number of positive cases of *beta-catenin* and its intensity decreased as the cancer differentiation become poor. In our study, nine number of cases of the oral mucosal biopsies showed cytoplasmic/nuclear *beta-catenin* expression with all PDOSCC cases having low immunoreactivity score of 1-4. While, 78 number of cases showed membranous *beta-catenin* expression with all PEH cases having high immunoreactivity score of > 4. Our results are concordant to those of previous studies performed by Yu et al., (2005), who compared expression of *beta-catenin* in normal tissue to cancerous tissue and concluded that in regular ordinary tissue *beta-catenin* expression was high while in cancerous tissue its expression was low showing poor prognosis. 

Our current results are parallel to those of Japanese pathologist, (Yun et al., 2010), who studied high *Ki-67* expression and low *beta-catenin* expression which is significantly linked with poorly and moderately differentiated carcinoma (p<0.05). A point noteworthy is that the sample size of the current study is huge making the results more reliable and statistically significant. 

On the basis of our study, the present results can be interpreted that in OSCC, the entire thickness of epithelium showed nuclear staining with *Ki-67*, while in PEH there was increased nuclear staining only in the basal layer of the epithelium. On the other hand, number of positive cases and immunoreactive score of *beta-catenin* decreased as the grade of oral squamous cell carcinoma increases. 

Hence, we concluded that opposite expression of *Ki-67* and *beta-catenin* play a vital role in forecasting histological grades of differentiation in oral mucosal biopsies and prognosis of the lesion. Therefore, helping clinicians to arrive at a final diagnosis before planning any surgical management.

But because of small tissue estimate, the utility of this panel is constrained. One of the limitations of this study is that the size of mucosal biopsies is so tiny or small, if we want more sections of the tissue for further study; it is difficult to choose if the sufficient tissue is cleared out in the block. For this purpose, we had also learnt from our present study to slice the tissue fragment for immunohistochemical analysis at the same time when routine sections were sliced so that there is reduce shaving and we have more left over tissue. One of the greatest limitations of our present study is that the sample size of PDOSCC was scarce owing to the patient turnout in our set up. No follow ups were possible owing to the short duration of present study. 

## Approved By Any Scientific Body

An institutional ethical committee approval (IRB, AFIP) (letter no. MP-ORP16-10/READ-IRB/17/394) was taken before the start of the study. Later on, defence of thesis was taken and approved by external and internal supervisor appointed by the university after correction of minor mistakes. Thus, this article is the part of approved thesis with the same title.
